# 2-Chloro-7,8,9,10-tetra­hydro­cyclo­hepta­[*b*]indol-6(5*H*)-one

**DOI:** 10.1107/S1600536812022945

**Published:** 2012-05-26

**Authors:** R. Archana, E. Yamuna, K. J. Rajendra Prasad, A. Thiruvalluvar, R. J. Butcher, Sushil K. Gupta, Sema Öztürk Yildirim

**Affiliations:** a PG Research Department of Physics, Rajah Serfoji Government College (Autonomous), Thanjavur 613 005, Tamilnadu, India; b Department of Chemistry, Bharathiar University, Coimbatore 641 046, Tamilnadu, India; c Department of Chemistry, Howard University, 525 College Street NW, Washington, DC 20059, USA; d School of Studies in Chemistry, Jiwaji University, Gwalior 474 011, MP, India; e Chemistry Department, Howard University, Washington, DC 20059, USA; f Department of Physics, Faculty of Sciences, Erciyes University, 38039 Kayseri, Turkey

## Abstract

In the title mol­ecule, C_13_H_12_ClNO, the dihedral angle between the benzene and pyrrole rings is 1.38 (9)°. The cyclo­heptene ring adopts a distorted twist chair and sofa conformation. Inter­molecular N—H⋯O hydrogen bonds form an *R*_2_^2^(10) loop in the crystal packing. Further, weak C—H⋯O and C—H⋯π (involving the benzene ring) inter­actions are found in the crystal structure.

## Related literature

For the biological activity of indole derivatives, see: Gribble (2000 ▶); Knölker & Reddy (2002 ▶); Kawasaki & Higuchi (2005 ▶); Bennasar *et al.* (1993 ▶); Hong *et al.* (2006 ▶); Lacoume *et al.* (1972 ▶); Joseph *et al.* (1998 ▶, 2000 ▶). For related crystallographic studies of cyclo­hept[*b*]indoles, see: Archana *et al.* (2010 ▶, 2011 ▶). For hydrogen-bond motifs, see: Bernstein *et al.* (1995 ▶).
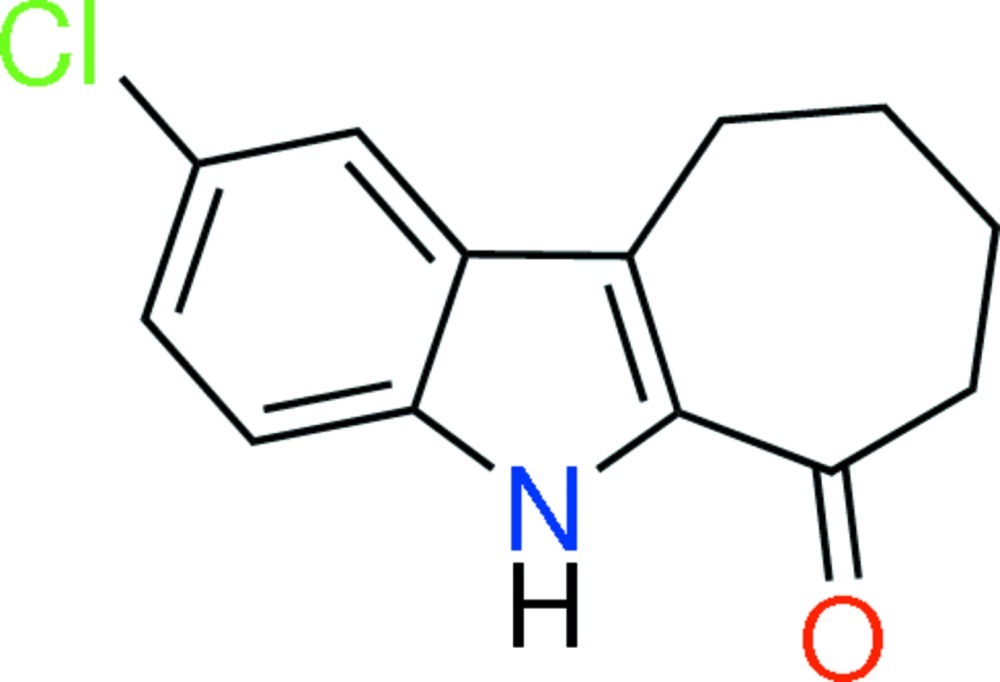


## Experimental

### 

#### Crystal data

C_13_H_12_ClNO
*M* *_r_* = 233.69
Monoclinic, 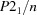

*a* = 11.6354 (4) Å
*b* = 6.3798 (2) Å
*c* = 14.4513 (5) Å
β = 92.767 (3)°
*V* = 1071.49 (6) Å^3^
*Z* = 4
Mo *K*α radiation
μ = 0.33 mm^−1^
*T* = 150 K
0.40 × 0.30 × 0.30 mm

#### Data collection

Agilent Xcalibur Ruby Gemini diffractometer
Absorption correction: multi-scan *CrysAlis PRO* (Agilent, 2011 ▶) *T*_min_ = 0.879, *T*_max_ = 0.907
4977 measured reflections
2274 independent reflections
1836 reflections with *I* > 2σ(*I*)
*R*_int_ = 0.026

#### Refinement

*R*[*F* ^2^ > 2σ(*F* ^2^)] = 0.039
*wR*(*F* ^2^) = 0.096
*S* = 1.04
2274 reflections
149 parameters
H atoms treated by a mixture of independent and constrained refinement
Δρ_max_ = 0.27 e Å^−3^
Δρ_min_ = −0.26 e Å^−3^

### 

Data collection: *CrysAlis PRO* (Agilent, 2011 ▶); cell refinement: *CrysAlis PRO*; data reduction: *CrysAlis PRO*; program(s) used to solve structure: *SHELXS97* (Sheldrick, 2008 ▶); program(s) used to refine structure: *SHELXL97* (Sheldrick, 2008 ▶); molecular graphics: *ORTEP-3* (Farrugia, 1997 ▶) and *PLATON* (Spek, 2009 ▶); software used to prepare material for publication: *PLATON*.

## Supplementary Material

Crystal structure: contains datablock(s) global, I. DOI: 10.1107/S1600536812022945/tk5099sup1.cif

Structure factors: contains datablock(s) I. DOI: 10.1107/S1600536812022945/tk5099Isup2.hkl

Supplementary material file. DOI: 10.1107/S1600536812022945/tk5099Isup3.cdx

Supplementary material file. DOI: 10.1107/S1600536812022945/tk5099Isup4.cml

Additional supplementary materials:  crystallographic information; 3D view; checkCIF report

## Figures and Tables

**Table 1:** Hydrogen-bond geometry (Å, °) *Cg*2 is the centroid of the C1–C4,C4*A*,C10*B* ring.

*D*—H⋯*A*	*D*—H	H⋯*A*	*D*⋯*A*	*D*—H⋯*A*
N5—H5⋯O6^i^	0.837 (19)	2.13 (2)	2.904 (2)	153.2 (19)
C9—H9*A*⋯O6^ii^	0.99	2.55	3.228 (2)	125
C7—H7*A*⋯*Cg*2^iii^	0.99	2.95	3.7969 (19)	144

Symmetry codes: (i) 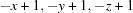; (ii) 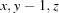; (iii) 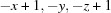.

**Fig. 1.**
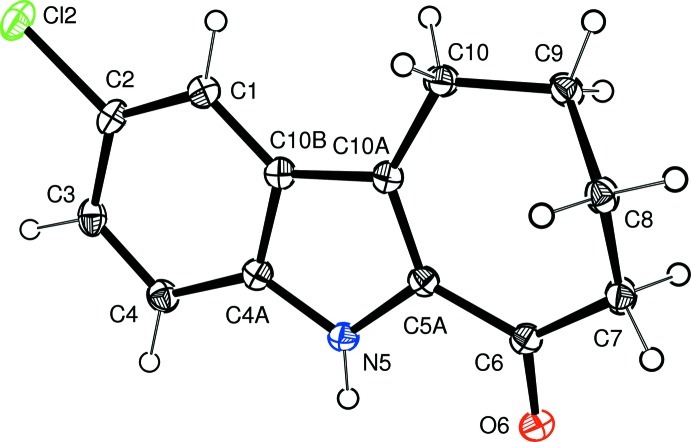
The molecular structure of the title compound, with displacement ellipsoids drawn at the 30% probability level. H atoms are shown as small spheres of arbitrary radius.

**Fig. 2.**
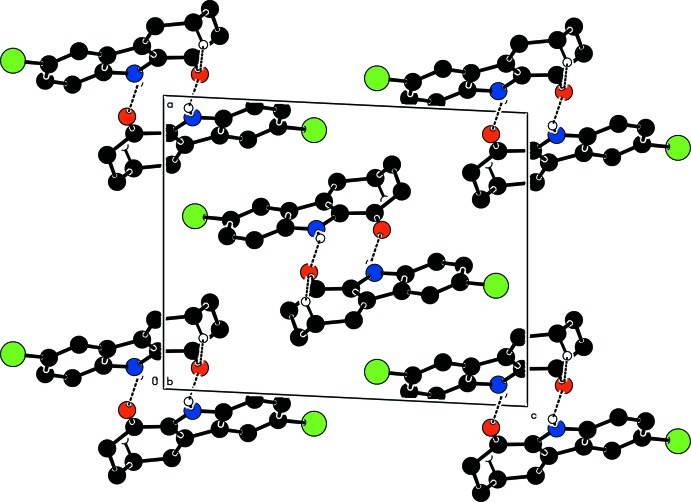
The crystal structure of (I), viewed down the b axis, showing the formation of a R22(10) ring by N—H···O hydrogen bonding (dashed lines). H atoms not involved in hydrogen bonding have been omitted.

## supplementary crystallographic information

### Comment

An indole nucleus coupled with prenylated indoles, carbazoles, indoloquinoline and cyclohept[*b*]indole alkaloids show high levels of biological activities including anti-fungal, anti-bacterial, anti-tumour and anti-HIV activities, as well as DNA interaction properties (Gribble, 2000; Knölker & Reddy, 2002; Kawasaki & Higuchi, 2005; Bennasar *et al.*, 1993; Hong *et al.*, 2006; Lacoume *et al.*, 1972; Joseph *et al.*, 1998; Joseph *et al.*, 2000). Recently, we reported related crystallographic studies for some cyclohept[*b*]indoles from our laboratory (Archana *et al.*, 2010; Archana *et al.*, 2011).

In the title molecule, Fig. 1, the dihedral angle between the benzene and pyrrole rings is 1.38 (9)°. The cycloheptene ring adopts a distorted twist chair and sofa conformation. Intermolecular N5—H5···O6 hydrogen bonds form a *R*^2^_2_(10) (Bernstein *et al.*, 1995) rings in the crystal structure. A weak C9—H9A···O6 intermolecular hydrogen bond along with a C7—H7A···π interaction, involving the benzene (C1–C4,C4A,C10B) ring, are also found in the crystal structure, Fig. 2 and Table 1.

### Experimental

A solution of 2-(2-(4-chlorophenyl)hydrazono)cycloheptanone (0.486 g, 0.001 mol) in a mixture of acetic acid (20 ml) and hydrochloric acid (5 ml) was refluxed on an oil bath pre-heated to 398 K for 2 h. The contents were then cooled and poured onto cold water with stirring. The brown solid which was separated by passing through a column of silica gel and eluted with (98:2, *v*/*v*) petroleum ether-ethyl acetate mixture yielded the title compound (0.167 g, 72%). This was recrystallized from ethanol.

### Refinement

The N—H atom was located in a difference Fourier map and refined freely. Other H atoms were positioned geometrically and allowed to ride on their parent atoms, with C—H = 0.95–0.99 Å and *U*_iso_(H) = 1.2*U*_eq_(parent atom).

### Crystal data

**Table:**

C_13_H_12_ClNO	*F*(000) = 488
*M**_r_* = 233.69	*D*_x_ = 1.449 Mg m^−^^3^
Monoclinic, *P*2_1_/*n*	Melting point: 389 K
Hall symbol: -P 2yn	Mo *K*α radiation, λ = 0.71073 Å
*a* = 11.6354 (4) Å	Cell parameters from 2495 reflections
*b* = 6.3798 (2) Å	θ = 3.2–28.4°
*c* = 14.4513 (5) Å	µ = 0.33 mm^−^^1^
β = 92.767 (3)°	*T* = 150 K
*V* = 1071.49 (6) Å^3^	Block, colourless
*Z* = 4	0.40 × 0.30 × 0.30 mm

### Data collection

**Table:**

Agilent Xcalibur Ruby Gemini diffractometer	2274 independent reflections
Radiation source: Enhance (Mo) X-ray Source	1836 reflections with *I* > 2σ(*I*)
Graphite monochromator	*R*_int_ = 0.026
Detector resolution: 10.5081 pixels mm^-1^	θ_max_ = 28.4°, θ_min_ = 3.5°
ω scans	*h* = −14→15
Absorption correction: multi-scan *CrysAlis PRO* (Agilent, 2011)	*k* = −8→7
*T*_min_ = 0.879, *T*_max_ = 0.907	*l* = −12→19
4977 measured reflections	

### Refinement

**Table:**

Refinement on *F*^2^	Primary atom site location: structure-invariant direct methods
Least-squares matrix: full	Secondary atom site location: difference Fourier map
*R*[*F*^2^ > 2σ(*F*^2^)] = 0.039	Hydrogen site location: inferred from neighbouring sites
*wR*(*F*^2^) = 0.096	H atoms treated by a mixture of independent and constrained refinement
*S* = 1.04	*w* = 1/[σ^2^(*F*_o_^2^) + (0.0373*P*)^2^ + 0.4689*P*] where *P* = (*F*_o_^2^ + 2*F*_c_^2^)/3
2274 reflections	(Δ/σ)_max_ = 0.001
149 parameters	Δρ_max_ = 0.27 e Å^−^^3^
0 restraints	Δρ_min_ = −0.26 e Å^−^^3^

### Special details

**Table:**

Geometry. Bond distances, angles *etc*. have been calculated using the rounded fractional coordinates. All su's are estimated from the variances of the (full) variance-covariance matrix. The cell e.s.d.'s are taken into account in the estimation of distances, angles and torsion angles
Refinement. Refinement of *F*^2^ against ALL reflections. The weighted *R*-factor *wR* and goodness of fit *S* are based on *F*^2^, conventional *R*-factors *R* are based on *F*, with *F* set to zero for negative *F*^2^. The threshold expression of *F*^2^ > 2σ(*F*^2^) is used only for calculating *R*-factors(gt) *etc*. and is not relevant to the choice of reflections for refinement. *R*-factors based on *F*^2^ are statistically about twice as large as those based on *F*, and *R*- factors based on ALL data will be even larger.

### Fractional atomic coordinates and isotropic or equivalent isotropic displacement parameters (Å^2^)

**Table:**

	*x*	*y*	*z*	*U*_iso_*/*U*_eq_	
Cl2	0.59361 (4)	−0.24239 (8)	0.08648 (3)	0.0357 (2)	
O6	0.57812 (10)	0.3697 (2)	0.60006 (8)	0.0280 (4)	
N5	0.56976 (12)	0.2648 (3)	0.42004 (10)	0.0225 (5)	
C1	0.63668 (14)	−0.1524 (3)	0.26774 (12)	0.0244 (5)	
C2	0.58717 (14)	−0.0838 (3)	0.18511 (12)	0.0262 (5)	
C3	0.53084 (14)	0.1108 (3)	0.17503 (12)	0.0275 (6)	
C4	0.52189 (15)	0.2416 (3)	0.24985 (12)	0.0256 (5)	
C4A	0.56937 (14)	0.1720 (3)	0.33521 (12)	0.0223 (5)	
C5A	0.62720 (14)	0.1378 (3)	0.48468 (11)	0.0211 (5)	
C6	0.63461 (14)	0.2152 (3)	0.58029 (12)	0.0229 (5)	
C7	0.71201 (15)	0.1148 (3)	0.65476 (12)	0.0273 (5)	
C8	0.80230 (15)	−0.0440 (3)	0.62766 (12)	0.0258 (5)	
C9	0.75353 (15)	−0.2421 (3)	0.58213 (12)	0.0254 (5)	
C10	0.73457 (16)	−0.2246 (3)	0.47726 (12)	0.0254 (5)	
C10A	0.66477 (13)	−0.0419 (3)	0.44109 (12)	0.0214 (5)	
C10B	0.62718 (14)	−0.0218 (3)	0.34549 (12)	0.0220 (5)	
H1	0.67583	−0.28293	0.27228	0.0293*	
H3	0.49882	0.15197	0.11609	0.0330*	
H4	0.48492	0.37398	0.24388	0.0307*	
H5	0.5389 (17)	0.380 (3)	0.4311 (14)	0.031 (6)*	
H7A	0.66192	0.04481	0.69880	0.0328*	
H7B	0.75270	0.22911	0.68916	0.0328*	
H8A	0.85431	0.02420	0.58455	0.0309*	
H8B	0.84908	−0.08411	0.68393	0.0309*	
H9A	0.67922	−0.27558	0.60929	0.0305*	
H9B	0.80688	−0.35980	0.59647	0.0305*	
H10A	0.81089	−0.21821	0.44992	0.0305*	
H10B	0.69653	−0.35477	0.45448	0.0305*	

### Atomic displacement parameters (Å^2^)

**Table:**

	*U*^11^	*U*^22^	*U*^33^	*U*^12^	*U*^13^	*U*^23^
Cl2	0.0376 (3)	0.0460 (3)	0.0233 (2)	0.0078 (2)	0.0001 (2)	−0.0094 (2)
O6	0.0322 (7)	0.0244 (7)	0.0275 (7)	0.0012 (5)	0.0032 (5)	−0.0036 (6)
N5	0.0242 (8)	0.0205 (8)	0.0229 (8)	0.0027 (7)	0.0009 (6)	−0.0004 (7)
C1	0.0213 (8)	0.0259 (9)	0.0261 (9)	0.0005 (8)	0.0008 (7)	−0.0003 (8)
C2	0.0241 (9)	0.0336 (10)	0.0209 (9)	−0.0007 (8)	0.0026 (7)	−0.0058 (8)
C3	0.0219 (9)	0.0382 (11)	0.0222 (9)	0.0016 (8)	0.0002 (7)	0.0042 (8)
C4	0.0231 (8)	0.0269 (10)	0.0267 (9)	0.0039 (8)	0.0013 (7)	0.0035 (8)
C4A	0.0182 (8)	0.0245 (9)	0.0242 (9)	−0.0027 (7)	0.0016 (7)	−0.0005 (8)
C5A	0.0187 (8)	0.0209 (9)	0.0235 (8)	−0.0033 (7)	0.0003 (6)	0.0007 (7)
C6	0.0224 (8)	0.0220 (9)	0.0245 (9)	−0.0069 (7)	0.0031 (7)	−0.0008 (7)
C7	0.0322 (9)	0.0287 (10)	0.0208 (9)	−0.0034 (8)	−0.0011 (7)	0.0004 (8)
C8	0.0255 (9)	0.0280 (10)	0.0234 (9)	−0.0028 (8)	−0.0037 (7)	0.0025 (8)
C9	0.0259 (9)	0.0229 (9)	0.0269 (9)	0.0003 (7)	−0.0039 (8)	0.0035 (8)
C10	0.0272 (9)	0.0222 (9)	0.0265 (9)	0.0021 (7)	−0.0023 (7)	−0.0013 (8)
C10A	0.0175 (8)	0.0236 (9)	0.0230 (9)	−0.0036 (7)	0.0005 (6)	−0.0005 (7)
C10B	0.0177 (8)	0.0244 (9)	0.0238 (8)	−0.0024 (7)	0.0009 (7)	0.0002 (8)

### Geometric parameters (Å, º)

**Table:**

Cl2—C2	1.7525 (19)	C8—C9	1.522 (3)
O6—C6	1.226 (2)	C9—C10	1.525 (2)
N5—C4A	1.361 (2)	C10—C10A	1.500 (3)
N5—C5A	1.384 (2)	C10A—C10B	1.435 (2)
N5—H5	0.837 (19)	C1—H1	0.9500
C1—C10B	1.407 (3)	C3—H3	0.9500
C1—C2	1.372 (2)	C4—H4	0.9500
C2—C3	1.408 (3)	C7—H7A	0.9900
C3—C4	1.374 (3)	C7—H7B	0.9900
C4—C4A	1.399 (2)	C8—H8A	0.9900
C4A—C10B	1.412 (3)	C8—H8B	0.9900
C5A—C6	1.466 (2)	C9—H9A	0.9900
C5A—C10A	1.389 (3)	C9—H9B	0.9900
C6—C7	1.512 (2)	C10—H10A	0.9900
C7—C8	1.524 (3)	C10—H10B	0.9900
			
C4A—N5—C5A	109.50 (16)	C1—C10B—C10A	133.14 (17)
C4A—N5—H5	124.8 (14)	C2—C1—H1	121.00
C5A—N5—H5	125.7 (14)	C10B—C1—H1	121.00
C2—C1—C10B	117.39 (17)	C2—C3—H3	120.00
Cl2—C2—C3	117.57 (13)	C4—C3—H3	120.00
Cl2—C2—C1	119.41 (14)	C3—C4—H4	121.00
C1—C2—C3	123.02 (17)	C4A—C4—H4	121.00
C2—C3—C4	120.45 (16)	C6—C7—H7A	107.00
C3—C4—C4A	117.35 (17)	C6—C7—H7B	107.00
N5—C4A—C4	129.78 (18)	C8—C7—H7A	107.00
N5—C4A—C10B	107.77 (15)	C8—C7—H7B	107.00
C4—C4A—C10B	122.44 (17)	H7A—C7—H7B	107.00
N5—C5A—C6	116.33 (16)	C7—C8—H8A	109.00
C6—C5A—C10A	134.44 (16)	C7—C8—H8B	109.00
N5—C5A—C10A	109.23 (15)	C9—C8—H8A	109.00
O6—C6—C7	118.84 (16)	C9—C8—H8B	109.00
C5A—C6—C7	122.28 (16)	H8A—C8—H8B	108.00
O6—C6—C5A	118.86 (16)	C8—C9—H9A	109.00
C6—C7—C8	119.54 (15)	C8—C9—H9B	109.00
C7—C8—C9	114.57 (15)	C10—C9—H9A	109.00
C8—C9—C10	113.70 (15)	C10—C9—H9B	109.00
C9—C10—C10A	116.94 (15)	H9A—C9—H9B	108.00
C5A—C10A—C10B	105.96 (15)	C9—C10—H10A	108.00
C10—C10A—C10B	122.67 (16)	C9—C10—H10B	108.00
C5A—C10A—C10	131.32 (16)	C10A—C10—H10A	108.00
C4A—C10B—C10A	107.53 (16)	C10A—C10—H10B	108.00
C1—C10B—C4A	119.33 (16)	H10A—C10—H10B	107.00
			
C5A—N5—C4A—C4	−180.00 (18)	N5—C5A—C6—C7	−168.77 (16)
C5A—N5—C4A—C10B	0.65 (19)	C10A—C5A—C6—O6	−169.76 (18)
C4A—N5—C5A—C6	−179.61 (15)	C10A—C5A—C6—C7	12.0 (3)
C4A—N5—C5A—C10A	−0.2 (2)	N5—C5A—C10A—C10	177.14 (17)
C10B—C1—C2—Cl2	−178.65 (13)	N5—C5A—C10A—C10B	−0.37 (19)
C10B—C1—C2—C3	1.6 (3)	C6—C5A—C10A—C10	−3.6 (3)
C2—C1—C10B—C4A	−0.8 (2)	C6—C5A—C10A—C10B	178.93 (18)
C2—C1—C10B—C10A	178.53 (18)	O6—C6—C7—C8	−166.26 (16)
Cl2—C2—C3—C4	179.40 (14)	C5A—C6—C7—C8	12.0 (3)
C1—C2—C3—C4	−0.8 (3)	C6—C7—C8—C9	−63.1 (2)
C2—C3—C4—C4A	−0.8 (3)	C7—C8—C9—C10	88.01 (18)
C3—C4—C4A—N5	−177.70 (17)	C8—C9—C10—C10A	−52.9 (2)
C3—C4—C4A—C10B	1.6 (3)	C9—C10—C10A—C5A	11.6 (3)
N5—C4A—C10B—C1	178.59 (15)	C9—C10—C10A—C10B	−171.28 (16)
N5—C4A—C10B—C10A	−0.87 (19)	C5A—C10A—C10B—C1	−178.59 (18)
C4—C4A—C10B—C1	−0.8 (3)	C5A—C10A—C10B—C4A	0.75 (19)
C4—C4A—C10B—C10A	179.72 (16)	C10—C10A—C10B—C1	3.6 (3)
N5—C5A—C6—O6	9.5 (2)	C10—C10A—C10B—C4A	−177.02 (15)

### Hydrogen-bond geometry (Å, º)

Cg2 is the centroid of the C1–C4,C4A,C10B ring.

**Table:**

*D*—H···*A*	*D*—H	H···*A*	*D*···*A*	*D*—H···*A*
N5—H5···O6^i^	0.837 (19)	2.13 (2)	2.904 (2)	153.2 (19)
C9—H9*A*···O6^ii^	0.99	2.55	3.228 (2)	125
C7—H7*A*···*Cg*2^iii^	0.99	2.95	3.7969 (19)	144

Symmetry codes: (i) −*x*+1, −*y*+1, −*z*+1; (ii) *x*, *y*−1, *z*; (iii) −*x*+1, −*y*, −*z*+1.
